# A New Approach to Age-Period-Cohort Analysis Using Partial Least Squares Regression: The Trend in Blood Pressure in the Glasgow Alumni Cohort

**DOI:** 10.1371/journal.pone.0019401

**Published:** 2011-04-27

**Authors:** Yu-Kang Tu, George Davey Smith, Mark S. Gilthorpe

**Affiliations:** 1 Division of Biostatistics, Centre for Epidemiology & Biostatistics, University of Leeds, Leeds, United Kingdom; 2 Leeds Dental Institute, University of Leeds, Leeds, United Kingdom; 3 MRC Centre for Causal Analyses in Translational Epidemiology, School of Social and Community Medicine, University of Bristol, Oakfield House, Oakfield Grove, Bristol, United Kingdom; University of Modena and Reggio Emilia, Italy

## Abstract

Due to a problem of identification, how to estimate the distinct effects of age, time period and cohort has been a controversial issue in the analysis of trends in health outcomes in epidemiology. In this study, we propose a novel approach, partial least squares (PLS) analysis, to separate the effects of age, period, and cohort. Our example for illustration is taken from the Glasgow Alumni cohort. A total of 15,322 students (11,755 men and 3,567 women) received medical screening at the Glasgow University between 1948 and 1968. The aim is to investigate the secular trends in blood pressure over 1925 and 1950 while taking into account the year of examination and age at examination. We excluded students born before 1925 or aged over 25 years at examination and those with missing values in confounders from the analyses, resulting in 12,546 and 12,516 students for analysis of systolic and diastolic blood pressure, respectively. PLS analysis shows that both systolic and diastolic blood pressure increased with students' age, and students born later had on average lower blood pressure (SBP: −0.17 mmHg/per year [95% confidence intervals: −0.19 to −0.15] for men and −0.25 [−0.28 to −0.22] for women; DBP: −0.14 [−0.15 to −0.13] for men; −0.09 [−0.11 to −0.07] for women). PLS also shows a decreasing trend in blood pressure over the examination period. As identification is not a problem for PLS, it provides a flexible modelling strategy for age-period-cohort analysis. More emphasis is then required to clarify the substantive and conceptual issues surrounding the definitions and interpretations of age, period and cohort effects.

## Introduction

One longstanding problem and controversy in observational research, such as epidemiology and sociology, surrounds how to estimate the distinct impacts of age, time period, and cohort on the changes in, for example, attitudes, behaviors and health outcomes in the population [1]–[7]. Due to the intrinsic mathematical relationship amongst the three variables, i.e. age + cohort  =  period, there is an identification problem in traditional regression analysis [8]. For example, suppose researchers observe an increasing trend in the incidences of the type-I diabetes in children in a geographic area over the last three decades [9], they hypothesize that this trend might be due to (1) the improved diagnostic skills in early indentifying young patients (i.e. time period effect), or (2) the decreased early infections due to improved hygiene and living environment (cohort effect), or maybe both. However, as the risk of the type-I diabetes also increases with age, to separate the effects of period and cohort, age too has to be accounted for. Since the three variables are mathematically related and have only two degrees of freedom, one has to be removed; otherwise, mathematical computation in the regression analysis cannot proceed, because the data matrix containing the three variables is not full-rank [10]. Mathematically speaking, a matrix without full rank is not invertible, and as a result, it makes the estimation of unique regression coefficients impossible without imposing additional constraints [11]. From a conceptual viewpoint, since one variable is the sum of the other two, it seems to makes little sense to estimate the “independent” effect of one by holding the other two fixed [12].

There have been many attempts to overcome this estimation (identification) problem in age-period-cohort analysis [1]–[8]. One common approach is to put constraints in the estimation process to overcome the computational problem with insufficient rank in the data matrix. While this type of modelling strategy produces simultaneous estimates of age, period, and cohort effects, it has been criticized in the statistical literature because the results are sensitive to the constraint chosen, and there is no empirical way to confirm the validity of the chosen constraints [2], [3], [8]. For instance, suppose in our previous hypothetical example of childhood type-I diabetes, the age of children is categorised into 3 groups: year 0 to 5, 5 to 9 and 10 to 14; time period is categorised into 5 groups: 1981 to 1985, 1986 to 1990, 1991 to 1995, 1996 to 2000, and 2001–2005; and cohort into 7 categories: 1971 to 1975, 1976 to 1980, 1981 to 1985, 1986 to 1990, 1991 to 1995, 1996 to 2000, and 2001–2005. As a result, there are 2, 4 and 6 dummy variables for age, period and cohort effects by using the first group for each as the reference. To investigate fully all effects, 12 dummy variables should be entered into the regression model simultaneously, but due to collinearity, we have to omit one. If, for example, the dummy variable for cohort born in 1976 to 1980 is omitted, this is equivalent to constraining its regression coefficient to zero [2], [8]. Apparently, there are at least 11 other constraints to be chosen (e.g. the dummy variable for cohort born in 1981 to 1985 is omitted instead), and each will yield slightly different results. However, it should be noted that to estimate the linear effects, certain constraints have to be imposed in the estimation of coefficients, and therefore the challenge is to seek for constraints that are justifiable and interpretable.

Another approach is to construct higher-order variables for those with perfect collinearity [13]. For instance, whilst age, period and cohort are perfectly collinear, age, period and the product term of age multiplied by cohort are not. However, even with just three variables, there are quite a few second-order variables to be tested, and the interpretation of these second-order effects is not straightforward. There are many other attempts in the literature to tackle the identification problem, but they do not always yield similar results, and some modelling strategies are very complex [2]–[8], [13]–[16].

Although traditional regression analysis (so-called generalised linear modelling) implemented in statistical software packages requires that the data matrix for covariates is full-rank, this is not a requirement for statistical methods for data dimension reduction, such as principal component regression and partial least squares (PLS) regression [17]–[22]. Therefore, collinearity and related identification problems are no longer a computational issue for these methods. The aim of this study is to demonstrate how to use PLS to separate the effects of age, period, and cohort, and to explain how PLSR provides a solution to the identification problem. A previous study used data from students who attended the Glasgow University between the years 1948 and 1968, and showed that systolic blood pressure (SBP) and diastolic blood pressure (DBP) were lower in students born in the 1940s than those born in the 1930s and 1920s [23]. In this study, we re-analyse this dataset using PLS to estimate the separate effects of age (age at examination), time period (year of examination), and cohort (year of birth) on blood pressure to both illustrate the methodology and seek what additional insight this provides.

## Methods

### Glasgow Alumni cohort

Details of the Glasgow Alumni cohort have been described elsewhere [23], [24]. Briefly, students attending Glasgow University between 1948 and 1968 were invited to participate in a health screening, including a questionnaire and clinical examination. Data collected included socio-economic background, health behaviours, and medical history. Height, weight, and blood pressure were also recorded. A total of 15,322 students (11,755 men and 3,567 women) participated in the study. Students born before 1925 or aged over 25 years at examination were excluded from the analyses. Two students were excluded because of data entry errors. Participants with missing values in the birth year and any of following confounders were also excluded: body height, body mass index, father's socio-economic background and cigarette smoking. Adjusted systolic blood pressure (SBP) and diastolic blood pressure (DBP) were obtained in 9,337 and 9,314 men, respectively, with the adjustment of all confounders. Similarly, adjusted SBP and DBP were obtained in 3,211 and 3,204 women, respectively.

Record linkage and follow-up of the Glasgow Alumni Cohort was under the ethics approval by the Multicentre Research Ethics Committees in the UK: MREC/99/0/9, “Influence of early life nutritional status, adolescent and adult diet on cancer incidence and mortality: a retrospective cohort study of Glasgow University students”, approved in March 2000. There was no consent collected at the time, as it is a historical cohort started 60 years ago; but this deemed acceptable by the ethics committees, if data used in an anonymised form - as they are throughout the analysis.

### Partial least squares (PLS) regression

PLS seeks to select components **t** that maximise the covariance between the outcome (SBP or DBP in this study) and **t** [20]–[22]. For *p* variables, *x*_1_, *x*_2_,…, *x_p_*, each PLS component **t** *_i_*, is a weighted composite of *p* covariates:
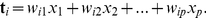
(1)Like principal component analysis (PCA), variables with small variances are penalised in the extraction of **t**, and therefore *x*_i_ in equation (1) is usually scaled to have unit variance and zero mean.

In contrast to PCA, PLS extracts components by taking into account their relationships with the outcomes. In PCA, the extraction of components is independent of the outcome variables, whereas in PLS, components are extracted explicitly for their association with the outcomes. The extraction of PLS components operates under the same constraints as with PCA: (i) the sum of the squared weights is unity, i.e. 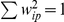; and (ii) the correlations amongst all components are zero. The number of **t** *_i_* that can be extracted is equal to the dimension (i.e. the rank) in the covariate matrix consisting of *x_i_*. For instance, in this study, there are only two dimensions in the data matrix consisting of age, the year at examination, and the year of birth; consequently, only two PLS components can be extracted from the three variables.

PLS components are ordered according to the amount of variance in the outcome that is explained by them, i.e. the first PLS component has greater covariance with the outcome than the second PLS component, and the second has greater covariance than the third, etc. In PLS, the first PLS component explains most of the outcome variance.

The PLS regression coefficient for each *x_i_* is then derived from the sum of products of the regression coefficients for PLS components and the weight for each *x_i_*. For example, when the outcome *SBP* is regressed on the two PLS components, the equation is given as:
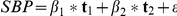


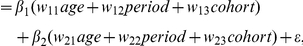
where *β*_1_ and *β*_2_ are the regression coefficients for PLS components 1 and 2, respectively, and *ε* is the residual error term. The PLS regression coefficient for *age* is therefore 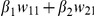.

Note that if all PLS components are used as new covariates, the results from the PLS regression, such as regression coefficients and *R* ^2^, are equivalent to those from PCA regression (and also ordinary least square regression, when the covariate matrix is full-rank). The advantage of PLS over PCA is that the first few components explain most of the covariance between the outcome and covariates.

### PLS and perfect collinearity

From a mathematical perspective, identification is a problem for the age-period-cohort analysis using ordinary least squares regression and related methods, because the inverse of the covariate matrix does not exist. However, whilst the inverse of a matrix without full rank does not exist, for a matrix without full rank, a mathematical technique, namely singular value decomposition (SVD), can still be used to obtain unique components of original variables, which are weighted compositions of original covariates [25]–[27]. In short, PCA is related to SVD of correlation/covariance matrix for the covariates, whilst PLS with one outcome is related to successive SVD of the vector for the correlations/covariances between the outcome and covariates [28]. This is why PCA and PLS have been widely used in bioinformatics where the number of variables exceeds the number of observations (which also gives rise to identification problem) [19], [22]. In PCA, three collinear variables with two dimensions (such as age, period, and cohort) are projected into two new latent variables, which are linear combinations of the original three variables; these new latent variable are then used as covariates for the regression analysis [23]. PLS may be viewed as a variant of PCA, where the two latent variables are rotated so that the first latent variable has the largest covariance with the outcome [29]. A technical explanation about how PCA and PLS work for perfectly collinear variables can be found in the Appendix S1. Briefly, it is well known that a linear model with a non-full rank covariate matrix (also called design matrix) has an infinite number of solutions for the choice of regression coefficients, and a constraint is therefore necessary to obtain a unique solution [30]–[32]. PLS implicitly imposes an inherent constraint in its algorithms that “naturally” arises from the intrinsic mathematical relationship: Age + Cohort  =  Period. The application of SVD effectively “inherits” this constraint in the estimation of the PLS regression coefficients [33], [34]. PLS does not intentionally impose this constraint; it emerges only due to the mathematical relationship of APC data. It can be shown that the imposed constraint is different when original or scaled variables are used in PLS, giving rise to different results. It is our view that the implicit constraint made by PLS regression seems to be a reasonable one, as it is a natural consequence of the intrinsic mathematic relationship amongst age, cohort and period. More explanation is found in the online Appendix S1.

### Selection of PLS component

To employ PLS is to maximise the covariance between the outcome and new composites, so it is justifiable to use the increments in the explained variance in the outcome (e.g. changes in R^2^) as a criterion for selecting PLS components. This gives us a measure of predictive ability, the predictive residual error sum of squares (PRESS) [35], [36]. To obtain this, the data are first split into a number of groups. For each, a prediction is obtained using the model derived from all other groups. For example, one observation is left out of the model, and we use the remaining observations to predict the outcome. PRESS is calculated as the sum of squares of the differences between the prediction for each observation (when it is left out of the model) and the observed value of the dependent variables.

### Data analysis

We first undertook sex-specific linear PLS regression for SBP and DBP by including the age at examination (16 to 25), the year of examination (1948 to 1968) and the year of birth (1925 to 1950) as continuous covariates. As PLS penalizes variables with small variances (e.g. age at examination), covariates are scaled to have unit variance [37]–[39]. Restricted cubic splines PLSR was then undertaken to explore nonlinear associations [40]. Three knots were placed for the year at examination (year 1954, 1959 and 1964) and four knots for the year of birth (year 1930, 1935, 1940, 1945 and 1950). In the final analysis, we created dummy variables for the three continuous variables to compare the results to those from linear and restricted cubic splines PLS regression. No arbitrary constraint on the dummy variables was required for PLS regression. All analyses were undertaken using a free data-mining software Tanagra (version 1.4.36, http://chirouble.univ-lyon2.fr/~ricco/tanagra/en/tanagra.html) with 1000 nonparametric bootstraps to obtain 95% confidence intervals.

## Results

Tables 1 and 2 show the adjusted mean blood pressure stratified by the year of examination or the year of birth for men and women, respectively. In general, participants born in the 1920s went to university slightly older than those born later. There seemed to be a decreasing trend in blood pressure for both the year of birth and the year at examination.

**Table 1:** Mean adjusted systolic blood pressure (SBP), diastolic blood pressure (DBP) and age at examination (Age) for men in Glasgow Alumni cohort according to their year of birth (Birthyear) and year of examination (Examyr).

		SBP		DBP		Age	
Birth year	N	Mean	SD	Mean	SD	Mean	SD
1925	240	107.7	11.9	72.3	7.7	24.1	0.6
1926	332	109.7	12.3	73.5	8.4	23.3	0.9
1927	352	108.4	12.7	71.9	8.8	22.5	1.0
1928	385	107.9	12.8	71.2	7.8	21.8	1.2
1929	447	107.8	12.1	70.7	8.1	20.9	1.4
1930	491	108.2	11.4	71.2	8.0	20.1	1.7
1931	523	108.2	12.5	70.1	7.9	19.7	1.8
1932	457	108.6	12.4	69.4	8.2	19.7	1.9
1933	461	106.4	11.5	69.1	8.3	19.8	2.0
1934	436	106.6	12.9	68.1	7.8	19.9	1.9
1935	379	105.9	13.4	67.7	8.7	19.9	1.8
1936	426	106.9	12.7	67.2	8.7	19.7	1.8
1937	449	106.8	13.6	68.0	10.2	19.4	1.8
1938	445	106.6	12.4	67.1	8.8	19.2	1.8
1939	402	105.9	12.8	67.0	8.5	19.3	1.8
1940	356	107.0	12.5	67.3	8.5	19.5	2.0
1941	327	106.5	13.4	67.4	8.2	19.6	2.0
1942	317	104.3	13.6	65.9	8.3	19.8	2.0
1943	416	105.6	13.8	66.4	8.9	19.7	1.9
1944	480	103.5	13.9	66.2	8.0	19.5	1.7
1945	477	102.8	12.6	65.9	8.7	19.2	1.5
1946	430	102.0	12.3	65.5	8.1	19.0	1.4
1947	446	101.0	12.5	66.2	7.8	18.9	1.2
1948	380	99.9	12.1	66.0	7.1	18.5	0.9
1949	247	99.2	12.6	64.4	8.3	18.0	0.7
1950	118	101.4	13.4	65.7	8.9	17.7	0.5

Exam year							
1948	315	107.1	12.2	71.9	8.4	20.2	1.9
1949	1,096	108.6	12.1	72.0	7.5	20.9	2.0
1950	759	109.1	12.6	71.4	8.6	21.0	2.2
1951	771	108.1	11.9	69.3	8.5	20.3	2.1
1952	437	107.7	12.6	68.6	7.8	19.9	2.1
1953	417	104.9	12.4	67.8	8.2	19.9	2.0
1954	229	107.6	12.4	67.9	9.5	19.3	2.1
1955	662	107.2	12.4	68.5	8.6	19.7	1.9
1956	493	107.9	13.5	69.0	10.6	19.6	2.0
1957	419	106.1	12.8	66.4	8.2	19.8	2.1
1958	454	105.1	12.4	67.2	8.5	19.7	1.9
1959	374	106.5	13.1	67.7	7.7	19.5	2.0
1960	252	107.3	12.7	68.3	8.3	19.9	2.0
1961	370	107.4	14.2	66.4	8.7	19.6	2.0
1962	385	104.8	13.6	66.1	8.2	19.2	1.7
1963	452	103.8	13.3	66.3	8.6	19.4	1.8
1964	514	101.7	12.5	64.6	8.2	19.2	1.6
1965	502	101.8	12.5	66.7	7.4	19.5	1.8
1966	523	100.8	12.2	66.7	7.9	19.2	1.9
1967	473	100.0	12.1	65.6	7.9	19.7	1.9
1968	322	100.4	13.5	65.3	8.9	20.0	1.7

**Table 2:** Mean adjusted systolic blood pressure (SBP), diastolic blood pressure (DBP) and age at examination (Age) for women in Glasgow Alumni cohort according to their year of birth (Birthyr) and year of examination (Examyr).

		SBP		DBP		Age	
Birth year	N	Mean	SD	Mean	SD	Mean	SD
1925	16	103.1	14.1	61.8	9.5	24.1	0.9
1926	19	104.6	11.7	58.3	8.8	23.0	0.8
1927	51	105.5	11.0	62.2	6.8	22.1	0.8
1928	95	103.0	11.5	60.5	7.1	21.5	1.1
1929	130	105.1	11.9	60.7	6.6	20.4	1.1
1930	159	106.1	11.8	61.3	7.5	19.4	1.2
1931	197	106.5	13.6	61.7	7.7	18.9	1.3
1932	202	107.2	12.3	60.9	7.8	18.8	1.3
1933	133	103.3	13.8	59.5	7.2	18.7	1.3
1934	168	100.9	12.1	57.7	8.1	18.7	1.2
1935	111	99.4	10.3	56.4	7.6	18.8	1.4
1936	128	100.9	11.8	58.5	7.3	18.9	1.6
1937	107	100.0	11.4	57.5	7.0	18.8	1.4
1938	149	98.3	11.3	56.5	7.5	18.7	1.6
1939	121	97.4	9.9	56.6	7.3	19.3	1.7
1940	120	98.6	10.3	57.7	7.1	19.2	1.6
1941	106	99.2	12.2	59.8	7.3	19.2	1.7
1942	124	98.2	11.0	57.6	8.5	19.7	1.8
1943	186	95.6	10.7	56.5	7.1	19.3	1.5
1944	173	95.6	10.1	56.3	7.0	19.4	1.6
1945	165	95.8	9.8	56.5	7.0	19.3	1.4
1946	185	97.6	11.9	57.4	6.9	19.2	1.5
1947	217	97.7	10.1	59.0	7.1	18.9	1.2
1948	182	97.6	10.5	58.6	6.6	18.6	0.9
1949	115	97.4	9.9	57.4	6.4	18.1	0.7
1950	59	94.6	11.5	55.6	7.0	17.6	0.6

Exam year							
1948	113	104.7	10.5	61.4	6.6	19.1	1.6
1949	293	104.8	11.4	60.7	7.7	19.6	1.7
1950	257	106.6	13.3	61.0	7.1	19.6	1.8
1951	236	108.0	13.7	61.6	8.1	19.2	1.6
1952	152	102.3	12.1	58.8	7.2	18.8	1.5
1953	128	98.2	10.2	57.0	7.9	19.2	1.8
1954	131	100.6	12.4	57.8	7.1	18.7	1.3
1955	127	99.5	10.4	56.7	7.0	18.6	1.6
1956	124	98.5	11.0	55.6	7.4	18.9	1.6
1957	72	97.8	10.2	58.6	7.0	19.3	1.7
1958	132	98.9	12.3	58.3	8.1	19.1	1.4
1959	138	100.1	10.9	58.4	7.8	19.2	1.5
1960	80	97.9	10.6	57.3	7.8	18.9	1.5
1961	135	98.1	11.3	57.8	6.7	18.9	1.4
1962	133	94.5	10.6	57.5	7.2	18.9	1.3
1963	170	97.0	10.3	54.8	7.1	19.3	1.6
1964	207	96.9	11.0	56.5	7.5	19.2	1.6
1965	201	97.2	9.6	58.4	6.7	19.2	1.8
1966	209	96.7	10.3	58.2	6.8	19.1	1.7
1967	236	95.7	10.1	57.9	6.8	19.3	1.5
1968	144	98.2	11.3	58.1	6.7	19.8	1.5

Table 3 shows the results from linear PLS analysis with one or two components. Whilst the PLS regression coefficients for age at examination (Age) differed slightly between the two models, there was little difference in the coefficients for the year of birth (Birthyear) and the year at examination (Examyear). Both Birthyear and Examyear showed similar negative associations with blood pressure in men and women. The R^2^ in the PLS model for SBP in men with one component was 3.45%, which is about 92% of the variance in SBP that could be explained by the three covariates. For the other models, the second component added little to the explained variance in blood pressure. Men born later in this cohort had lower SBP than those born earlier (−0.17 mmHg/per year, 95% Confidence Intervals [CI]: −0.18 to −0.15). Men who attended the university later had lower SBP than those who attended earlier (−0.2, 95%CI: −0.18 to 0.22). DBP for men born later was lower than that for those born earlier (−0.14, 95%CI: −0.15 to −0.13), and for those who attended the university later was 0.15 mmHg/per year lower than those who attended earlier. SBP for women born later was lower than for those born earlier (−0.25, 95%CI: −0.28 to −0.22). Women who attended the university later had lower SBP than those who attended earlier (−0.27, 95%CI: −0.30 to −0.24). Women who were born (or attended the university) later had lower DBP than those who were born (or attended the university) (−0.09 mmHg, 95%CI: −0.11 to −0.07) earlier.

**Table 3:** Results from linear partial least squares regression with scaled variables for men and women in Glasgow Alumni Cohort.

		Men				Women			
		1-Comp		2-Comp		1-Comp		2-Comp	
	Variables	Coef	95% CI	Coef	95% CI	Coef	95% CI	Coef	95% CI
**SBP**	**Age**	0.08	(0.02 to 0.14)	−0.28	(−0.40 to −0.14)	−0.02	(−0.15 to 0.12)	−0.30	(−0.55 to −0.05)
	**Birth year**	−0.17	(−0.18 to −0.15)	−0.17	(−0.19 to −0.15)	−0.25	(−0.28 to −0.22)	−0.25	(−0.28 to−0.22)
	**Exam year**	−0.20	(−0.22 to −0.18)	−0.24	(−0.27 to −0.22)	−0.27	(−0.30 to −0.24)	−0.28	(−0.31 to −0.25)
	**R^2^ (%)**	3.45		3.76		8.07		8.21	
**DBP**	**Age**	0.27	(0.23 to 0.31)	0.25	(0.16 to 0.33)	0.11	(0.03 to 0.19)	0.12	(−0.03 to 0.27)
	**Birth year**	−0.14	(−0.15 to −0.13)	−0.14	(−0.15 to −0.13)	−0.09	(−0.11 to −0.07)	−0.09	(−0.11 to −0.07)
	**Exam year**	−0.15	(−0.16 to −0.14)	−0.15	(−0.17 to −0.13)	−0.09	(−0.11 to −0.07)	−0.09	(−0.11 to −0.07)
	**R^2^** **(%)**	6.05		6.05		2.79		2.79	

**Figure 1:**
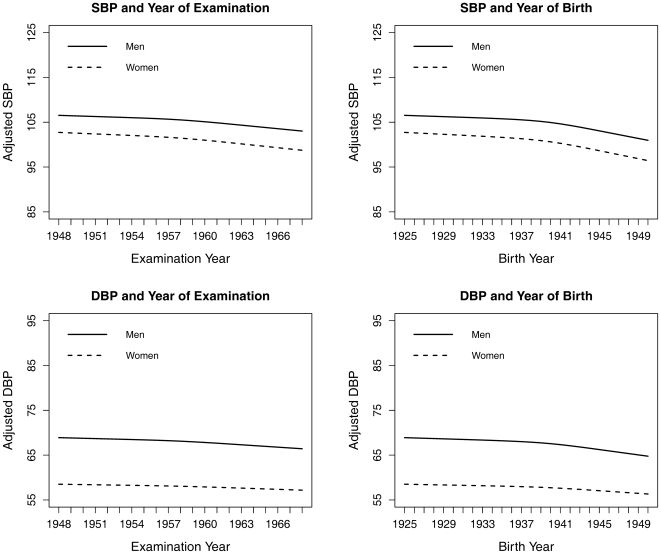
The relationship between adjusted blood pressure and year at birth or examination for men and women in the restricted cubic splines partial least squares regression. For SBP, the R^2^ is 3.75% for men and 4.48% for women, which are greater than 80% of total R^2^ that can be explained. For DBP, the R^2^ is 7.48% for men and 2.40% for women, which are greater than 56% of total R^2^ that can be explained.

The component selection statistic, PRESS, identified only one component for the restricted cubic splines PLS analysis for the associations of blood pressure with Birthyear and Examyear with Age entered as a continuous variable. Figure 1 shows that there were decreasing tends in the blood pressure for both variables in men and women. The trend for the relationships between blood pressure and Birthyear for men and women showed a slightly greater decline around year 1941.

**Figure 2:**
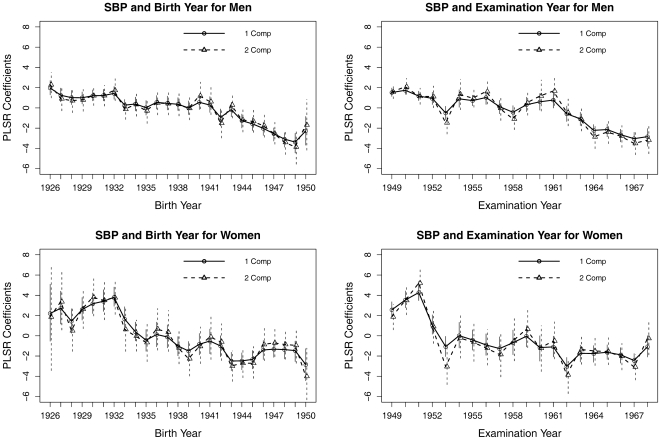
The partial least squares regression coefficients plots for the year of birth or the year at examination. Both variables and age at examination are treated as categorical with the first year as the reference group. The vertical bars are the confidence intervals. The outcome variable is systolic blood pressure (SBP). PRESS only selected one PLS component for each model. The two-component models explained almost all the variances (>98%) in blood pressure than can be explained.

**Figure 3:**
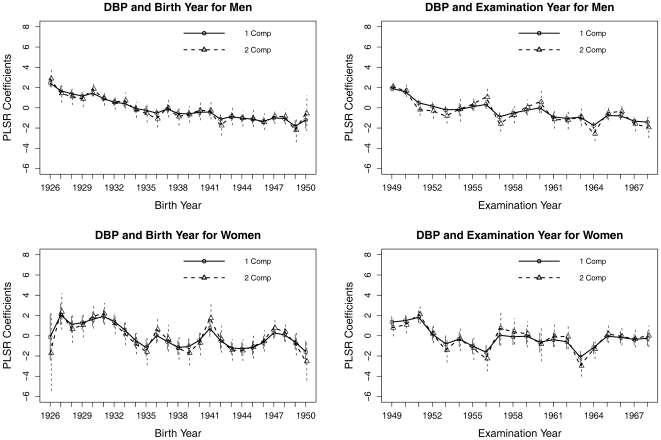
The partial least regression coefficients plots for the year of birth or the year at examination. Both variables are treated as categorical with the first year as the reference group. The vertical bars are the confidence intervals. The outcome variable is systolic blood pressure (SBP).

Figures 2 and 3 show the trends in SBP and DBP, respectively, when Birthyear and Examyear were treated as categorical variables. The decreasing trends were less notable in DBP than in SBP and less notable in women than in men. For men, the trend in SBP showed a small further decline around 1943 for Birthyear and around 1961 for Examyear, indicating both cohort and period effects.

## Discussion

The previous analysis of the Glasgow Alumni cohort found substantial downward trends in blood pressure occurred in male and female students after confounding factors were controlled for [19]. Results from our re-analysis using PLS are generally consistent with those from the previous analysis, showing a cohort effect on blood pressure. However, the previous analysis only adjusted for age at examination without considering the effect of the year at examination. Our study shows that there was also a negative trend in blood pressure for period effects, i.e. students who attended the university in the 1960s had lower blood pressure than those attending university in the 1950s.

### Interpretation of age, period and cohort effects in PLS analysis

Research aiming at solving the collinearity problem in age-period-cohort analysis has generated an extensive literature, and most approaches have tried to accommodate the collinearity problem within the scope of traditional regression analyses. From a statistical viewpoint, an additional constraint can be made to make the effects of age, period, and cohort estimable, but the problem is that there are too many potential constraints. Hence, the more pertinent issue is rather that of interpretation with regard to the chosen constraint. As explained in the Appendix S1, an implicit constraint is imposed in PLS estimation, and this corresponds to the intrinsic mathematic relationship amongst age, period, and cohort. We therefore feel that the constraint imposed by PLS is both justifiable and interpretable.

The cohort effect is usually attributed to the impact of early environment, such as nutrition in pregnancy and early childhood [5], [6]. According to the developmental origins of health and disease hypothesis, early growth environment may have an important impact on health outcomes in later life [41], [42]. When the foetus and infant makes predictive adaptive responses to the environment in the early developmental process, adaptations chosen to cope with the unfavourable environment may have adverse effects for health in later phases of the lifecourse. It has also been suggested that early childhood conditions, such as dehydration, may be associated with blood pressure in later life [43], [44]. On the other hand, changes in nutrition and diet, such as reduction in salt intake and increased consumption of vegetable and fruits in the first half of the last century in the UK [23], [45], may have contributed to the decreasing trend in blood pressure across the year of birth found in the previous and the present studies. Nevertheless, the negative associations between blood pressure and year of examination found in the present study also suggest that the improved nutrition and living environment in the UK might have a continuing impact on population health in adolescence and early adulthood.

In this study, we also found that there seemed to be differences in trends for DBP, where the decreasing trends in exam year and birth year were less notable in women than in men, but for SBP, men and women had similar trends. Women had on average had lower DBP than men by about 10 mmHg, and whilst healthy diets or other factors were associated with improved blood pressure, there might be a physiological limit on how much reduction in blood pressure can be attained due to such factors.

Many studies in the age-period-cohort analysis literature aim to resolve the identification problem in order to estimate the “unique” contribution of the three components [2]. However, as one recent study argued, the conceptual definitions of such effects are not always clear and therefore require further elaboration [5]. The cohort effect, such as that represented by the year of birth in this study, is usually interpreted as the effect of early life experience, e.g. early nutrition in epidemiological research. The period effect is interpreted as exposure or events in later life. From a lifecourse perspective, the impact of environment and its interactions with biological factors continues throughout the developmental process. The demarcation of lifecourse experience into different phases such as cohort versus period, or early versus later life, is a conceptual framework for research, but the underlying biological process is nevertheless continuous. Age, period and cohort are not only mathematically related but also conceptually connected. PLS analysis partitions their joint lifecourse effects according to their covariance structure with each other and the outcome. Results from PLS yield the partitioning of the *total* effects of age, period and cohort, which has meaning and utility. This is a similar idea to that suggested recently by O'Brien of the partitioning of the total variance [46]. Whilst it may be tempting to interpret the PLS regression coefficients as the “independent” contributions of age, period and cohort, it is more appropriate to view them as their “relative” importance in contribution. In view of this perspective of interpretation, cohort and period had similar effects on blood pressure in this study.

### Comparisons between PLS and other modelling strategies in the literature

The major difference between PLS and other modelling strategies is that it is straightforward to incorporate all perfectly collinear variables into the same model. Some approaches proposed in the literature can only be applied to aggregated data [3], [7], [8], but PLS can be used to analyse both individual data such as those in this study and aggregated data such as mortality rates for different age groups in different years. For example, a commonly used approach is to plot the trends in the outcome against age groups for different birth cohorts, and period effects are inferred from the differences in trends between cohorts [4]. PLS is therefore complementary to those approaches. For wider applications of PLS in epidemiology, further developments are required to extend PLS to generalised linear models [47]–[49]. Nevertheless, PLS already provides a flexible modelling strategy for age-period-cohort analysis.

### Concluding remarks

There is an extensive literature in epidemiology and social sciences as to how to estimate age, cohort and period effects. Whilst some of the debates and controversies focused upon the identification issue in the estimation [4]–[7], some are more concerned with the meaning and interpretation of those effects [1]–[3], [8]. In this study, we propose to use PLS to address the former, but whilst identification is no longer a computation issue for PLS, more effort is required to clarify the substantive and conceptual issues regarding the definitions and interpretations of age, period and cohort effects. Those conceptual questions may be even harder to answer.

## Supporting Information

Appendix S1 Supporting appendix
(DOC)
