# Supplementary material for: A New Approach to Age-Period-Cohort Analysis Using Partial Least Squares Regression: The Trend in Blood Pressure in the Glasgow Alumni Cohort
Source: PLoS One. 2011 Apr 27;6(4):e19401. doi: 10.1371/journal.pone.0019401 (PMC3083444; doi:10.1371/journal.pone.0019401)
Supplement: Appendix S1 — Supporting appendix (DOC) [file pone.0019401.s001.doc]

# Appendix

## The identification problem with the Age-Period-Cohort analysis

The aim of this Appendix is to explain how partial least squares regression resolves the identification issues caused by the intrinsic mathematical relationship in the age-period-cohort analysis. The citation numbers correspond to those in the reference list for the main paper.

As stated in the Introduction, a matrix without full rank is not invertible, and as a result, it makes the estimation of model coefficients impossible using ordinary least squares regression or generalized linear modeling. However, the problem is not that there is no solution to the model for an age-period-cohort analysis, rather there are too many (an infinite number of) solutions. For instance, the relationship between systolic blood pressure (*SBP*) and the three variables, *Age* (chronological age at examination), *Cohort* (year of birth) and *Period* (year at examination) in an ordinary least squares (OLS) regression is written as:

$SBP=b_{0}+b_{1}Age+b_{2}Cohort+b_{3}Period+e$, (A-1)

where *b*_0_ is the intercept, *b*_1_, *b*_2_ and *b*_3_ are the regression coefficients for *Age*, *Cohort*, and *Period*, respectively, and *e* is the residual error term. To simplify our discussion, we assume that all the four variables are centered, i.e. their means have been subtracted from initial individual values for each variable. Therefore, we can exclude the intercept in equation (A-1) from the model. In matrix notation, equation (A-1) can be expressed as:

$\mathbf{y}=X\mathbf{b}+\mathbf{e}$ (A-2)

where **y** is a vector for *SBP*, and *X* is the design matrix for *Age*, *Period*, and *Cohort*, **b** is a vector for *b*_1_, *b*_2_ and *b*_3_, and **e** is a vector for the residuals. The estimation for **b** is to solve the following equation:^27-29,46^

$\mathbf{b}=(X^{T}X)^{-1}X^{T}\mathbf{y}$ (A-3)

where *X^T^* is the transposed matrix of *X*, and (*X^T^X*)^-1^ is the inverse of *X^T^X*. The estimated *SBP* ($\hat{\mathbf{y}}$) is given as:

$\hat{\mathbf{y}}=X(X^{T}X)^{-1}X^{T}\mathbf{y}$. (A-4)

Statistical software packages cannot proceed with computation unless at least one of the three covariates is removed from the model, because the product matrix *X^T^X* is not full-rank and consequently (*X^T^X*)^-1^ does not exist.^27,29^ However, whilst (*X^T^X*)^-1^ does not exist, there are an infinite number of generalized inverse matrices for *X^T^X*, (*X^T^X*)^–^, and $\hat{\mathbf{y}}$ in equation (A-4) is not affected by the selection of (*X^T^X*)^–^, i.e. whilst (*X^T^X*)^–^ is not unique, $\hat{\mathbf{y}}$ is.^27-29,46^ When *X^T^X* is full-rank, it can be shown that (*X^T^X*)^–^ is unique and is equivalent to (*X^T^X*)^-1^.^27,46^  Therefore, the problem with the age-period-cohort analysis is that, although we can estimate the joint effect of *Age*, *Period*, and *Cohort*, we cannot estimate their individual effects, unless auxiliary information about the relationship amongst their effects on the outcome is also available. In statistical jargon, because of insufficient degrees of freedom, we need to impose a constraint on the estimation of the model specified by the equation (A-1) to obtain a unique **b**, and different constraints will lead to different **b**.^27-29,46^

### ***Partial Least Squares Regression (PLSR) and perfect collineari****ty*

Amongst the infinite number of generalized inverse matrices, one special and unique generalized inverse matrix of *X^T^X***,** known as the Moore-Penrose inverse, (*X^T^X*)^+^, has been widely used in statistics to resolve the identification problem.^23,24,27-30,46^ As (*X^T^X*)^+^ satisfies more mathematical conditions than generalized inverses, and it is unique for any singular matrix.^23,24,27,46^ The Moore-Penrose inverse is closely related to a matrix algebra technique known as singular value decomposition (SVD).^23,24,27,46^ It is well known that results from the use of the Moore-Penrose inverse is equivalent to those from principal components regression (PCR) and partial least squares (PLS) regression when the maximum number of components is retained.^18-20,33-35^

For the age-period-cohort analysis, obtaining any solution requires the imposition of a constraint in the estimation of **b** in equation (A-1), and whether or not the solution is meaningful depends upon the choice of constraint, i.e. the conditions imposed in the estimation. This principle applies in general to all the “solutions” proposed in the literature on the APC analysis. We therefore seek to explore the statistical conditions imposed by PLS.

In PCR, the extraction of components is independent of the outcome variable, i.e. the same components are extracted in the same order as new covariates, irrespective of the outcome. From a data reduction point of view, this is not always desirable if the aim is to find a parsimonious model for predicting the outcome, because sometimes principal components with large variances may have limited association with the outcome.^47^ This potential weakness is amended in PLSR, as the extraction of components in PLSR aims to maximize the covariance with the outcome under the same constraints for PCR, i.e. that $\left|\mathbf{w}_{i}\right|=1$ and $t_{i}\perp t_{j}$ (*i* ≠ *j*) (see the discussion following equation (1) in the main text).

The original algorithms for calculating PLS models proposed by Herman Wold are known as nonlinear iterative partial least squares (NIPALS).^16-19^ The algorithms start with a selection of a column in *Y* (the matrix for dependent variables), and then repeats a sequence of the following steps iteratively until the covariance between **u**, a weighted composite of dependent variables, *y_j_* (*j* = 1 to *q*) and **t**, a weighted composite of covariates, *x_i_* (*i* = 1 to *p*), is maximized:^33-35^

Step 1: set **u** to be the first column of *Y*

Step 2: **w** = *X***^T^u**/(**u^T^u**)

Step 3: Scale **w** to be of length one

Step 4: **t** =*X***w**

Step 5: **c** = *Y***^T^t**/(**t^T^t**)

Step 6: Scale **c** to be of length one

Step 7: **u** =*Y***c**

Convergence is tested on the percentage change in the length of **t**.^35^ When convergence has been reached, PLS regression coefficients are obtained by regressing **u** on **t**: *b_PLS_* = **u^T^t**/(**t^T^t**). Residual matrices are calculated as, *X* = *X* **– tp^T^**, where **p** = *X*^T^**t**/(**t**^T^**t**), and *Y* = *Y* **– tc^T^**; then they are used for finding the next component. Note that if there is only one independent variable **y**, **u** = **y** in Step 1, and **c** is a scalar in Step 5.

Although PLSR was first developed as a set of algorithms to extract components in an iterative process, it was shown that PLSR is related to a series of SVD of *X^T^***y**.^25,34,35^ Taking the association between *SBP* and *Age*, *Cohort* and *Period*, in men as an example, the matrix *X^T^***y** (where all four variables are centered) is:

$$X^{T}\mathbf{y}=\left[\begin{matrix} Age & Cohort & Period \end{matrix}\right]^{T}\left[SBP\right]=\left[\begin{matrix} 6536.517 \\ -149922.322 \\ -143385.825 \end{matrix}\right]$$

Astute readers may notice that 6536.517 + (-149922.342) = (-143385.825), i.e. the sum of the first and second elements is equal to the third, which corresponds to the simple mathematical relation *Age* + *Cohort* = *Period*.

**Proof:**

let us call *Age* *x*_1_, *Cohort* *x*_2_, *Period* *x*_3_ and *SBP* *y*, i.e. *x*_1_ + *x*_2_ = *x*_3_. After subtracting the mean from each variable, we find:

$(x_{1}-\overline{x_{1}})+(x_{2}-\overline{x_{2}})=(x_{3}-\overline{x_{3}})$; (A-5)

where $\overline{x_{1}}$, $\overline{x_{2}}$, and $\overline{x_{3}}$ are the means of *x*_1_, *x*_2_, and *x*_3_, respectively. Multiplying both sides of equation (A-5) by $(y-\overline{y})$ (where $\overline{y}$ is the mean of *y*), we obtain the equality observed in *X^T^***y**.

We then undertake singular value decomposition for *X^T^***y**:

$SVD(X^{T}\mathbf{y})=SVD\left(\left[\begin{matrix} 6536.517 \\ -149922.322 \\ -143385.825 \end{matrix}\right]\right)=\left[\begin{matrix} 0.0315 \\ -0.7223 \\ -0.6908 \end{matrix}\right]*207554.6*\left[1\right]$.

The singular value 207554.6 is the squared root of the sum of squares of each element in *X^T^***y**: (6536.517)^2^ + (-149922.342)^2^ + (-143385.825)^2^ = (207554.6)^2^. 207554.6 divided by 9336 (the sample size minus 1) is 22.23, and its square 494.25 is the sum of the variance of the three projected vectors of *X* on **y** (i.e. the Step 2 in the PLS algorithms), if we undertake the SVD analysis for *X^T^***yy*^T^****X* instead, the first singular value will be 494.25 and the other singular values are all zero.^25^

Note that the left singular vector [0.0315, -0.7223, -0.6908]^T^ contains the weights for the first PLS component (**t**_1_), i.e.:

$$\mathbf{t}_{1}=0.0315*Age+(-0.7223)*Cohort+(-0.6908)*Period$$

We also note that in the singular vector, the sum of the first two elements is equal to the third: 0.0315 + (-0.7223) = (-0.6908). When *SBP* is regressed on **t**_1_, the regression coefficient is 0.266, and the PLSR model with 1 component can be written as:

$$\mathbf{t}_{1}=0.266*[0.0315*Age+(-0.7223)*Cohort+(-0.6908)*Period]$$

$$=0.008*Age-0.192*Cohort-0.184*Period$$

Again, the PLSR coefficients *b*_1_, *b*_2_ and *b*_3_, also satisfy the simple mathematical relation *b*_1_ + *b*_2_ = *b*_3_ (i.e. 0.008 + (-0.192) = -0.184). Although PLS algorithms do not "select" this constraint for the estimation of the regression coefficients (i.e. *b*_1_ + *b*_2_ = *b*_3_), the intrinsic mathematical relationship amongst the three covariates nevertheless give rise to this constraint implicitly, i.e. SVD of a singular matrix corresponds to a restriction in the estimation of coefficients.^30,48^ In fact, such a constraint in the estimation of **b** has been explicitly proposed in the literature based on a geometric idea.^49^

It should be noted that this constraint also applies to PLS models with two components, and this can be verified in the Table S1, which shows results from PLS models with one or two components. As explained in the previous section on PLS algorithms, the second component aims to maximize the covariance between *X*^(2)^ = *X* **– tp^T^** and *Y*^(2)^ = *Y* **– tc^T^**. As **p** = *X*^T^**t**/(**t**^T^**t**),

$X^{(2)}=X-\frac{\mathbf{tt}^{T}}{(\mathbf{t}^{T}\mathbf{t})}X$,

and as **t** = *X***w**,

$X^{(2)}=X-\frac{X\mathbf{ww}^{T}X^{T}}{(\mathbf{w}^{T}X^{T}X\mathbf{w})}X$.

We know that **w** is the singular vector of *X^T^***y**, and the elements *w*_1_, *w*_2_ and *w*_3_ in **w** satisfy the simple mathematical relation that *w*_1_ + *w*_2_ = *w*_3_. As *x*_1_ + *x*_2_ = *x*_3_ in *X*, the new variables $x_{1}^{(2)}$, $x_{2}^{(2)}$, and $x_{3}^{(2)}$ in *X*^(2)^ will also satisfy $x_{1}^{(2)}+x_{2}^{(2)}=x_{3}^{(2)}$.

Because the extraction of the second PLS component is equivalent to undertaking SVD of (*X*^(2)^)*^T^***y^(2)^**, where **y**^(2)^ = **y – tc^T^**, the weights in the second PLS components will also satisfy that $w_{1}^{(2)}+w_{2}^{(2)}=w_{3}^{(2)}$. As a result, this constraint in estimation is a general one: for *p* variables, if *x*_1_ + *x*_2_ + … + *x_p_*_-1_ = *x_p_*, their regression coefficients *b*_1_, *b*_2_, …, *b_p_*_-1_, and *b_p_* will always satisfy *b*_1_ + *b*_2_ + …+ *b_p_*_-1_ = *b_p_*, for PLS models with any number of components. More detailed discussion about the relation between constraint in linear model estimation and variables relation in the design matrix can be found in references 25, 30 and 48.

**Table S1: results from PLS analysis with unscaled variables**

|  | **Men** | | | | **Women** | | | |
| --- | --- | --- | --- | --- | --- | --- | --- | --- |
|  | **1-Comp** | | **2-Comp** | | **1-Comp** | | **2-Comp** | |
| **SBP** |  |  |  |  |  |  |  |  |
| **Variables** | **Coef** | **95% CI** | **Coef** | **95% CI** | **Coef** | **95% CI** | **Coef** | **95% CI** |
| **Age** | 0.008 | (0.002 to 0.015) | -0.208 | (-0.299 to -0.118) | -0.001 | (-0.009 to 0.006) | -0.210 | (-0.372 to -0.048) |
| **Birth year** | -0.192 | (-0.213 to -0.171) | -0.103 | (-0.145 to -0.061) | -0.258 | (-0.289 to -0.227) | -0.158 | (-0.242 to -0.075) |
| **Exam year** | -0.184 | (-0.203 to -0.165) | -0.312 | (-0.368 to -0.255) | -0.259 | (-0.289 to -0.229) | -0.368 | (-0.458 to -0.279) |
| **R^2^ (%)** | 3.53 |  | 3.76 |  | 8.039 |  | 8.21 |  |
| **DBP** |  |  |  |  |  |  |  |  |
| **Age** | 0.029 | (0.025 to 0.033) | 0.160 | (0.100 to 0.220) | 0.006 | (0.002 to 0.011) | 0.079 | (-0.027 to 0.184) |
| **Birth year** | -0.174 | (-0.188 to -0.161) | -0.227 | (-0.255 to -0.199) | -0.097 | (-0.117 to -0.078) | -0.131 | (-0.185 to -0.078) |
| **Exam year** | -0.145 | (-0.157 to -0.133) | -0.067 | (-0.104 to -0.030) | -0.091 | (-0.110 to -0.072) | -0.053 | (-0.111 to 0.005) |
| **R^2^ (%)** | 5.87 |  | 6.05 |  | 2.738 |  | 2.79 |  |

# *Scaling of covariates*

One problem with the unscaled analysis, as just shown, is that it penalizes variables with smaller variance. For instance, *Age* (age at examination) in our study has the smallest variance (4.15), because it has only a range of 10 years; the variance of *Cohort* (birth years) is 47.4, and the variance of *Period* (examination years) 37.7. Consequently, whilst the effect of *Age* is underestimated, the estimated effect of *Cohort* and *Period* are exaggerated, because the different ranges of these three variables are arbitrary and simply due to how the data were collected. If we restricted the range of birth years, it would not change the range of *Age*. In order not to penalize variables with small variances, covariates are usually scaled to have unit variance in PLS and PCR, and this is equivalent to giving differential weighting in the constraint during the estimation process, i.e. the simple constraint that *b*_1_ + *b*_2_ = *b*_3_ becomes:

$w_{1}b_{1S}+w_{2}b_{2S}=w_{3}b_{3S}$; (A-6)

where *b*_1S_, *b*_2S_ and *b*_3S_, are PLS regression coefficients when covariates are scaled in the extraction of partial least square components. This is because the scaled variables no longer satisfy the simple relationship amongst *Age*, *Cohort* and *Period*. Since equation (A-5) shows

$(x_{1}-\overline{x_{1}})+(x_{2}-\overline{x_{2}})=(x_{3}-\overline{x_{3}})$,

we then divide both sides by *s*_1_*s*_2_*s*_3_ to obtain:

$$\left(\frac{1}{s_{2}s_{3}}\right)x_{1S}+\left(\frac{1}{s_{1}s_{3}}\right)x_{2S}=\left(\frac{1}{s_{1}s_{2}}\right)x_{3S}$$

where *x*_1S_, *x*_2S_ and *x*_3S_, are scaled *Age*, *Cohort* and *Period* with unit variances, and *s*_1_, *s*_2_ and *s*_3_ are the standard deviations of *Age*, *Cohort*, and *Period*, respectively. Consequently, the standardized PLS regression coefficients *β*_1S_, *β*_2S_ and *β*_3S_ for scaled variables satisfy the following equality:

$\left(\frac{1}{s_{2}s_{3}}\right)\beta_{1S}+\left(\frac{1}{s_{1}s_{3}}\right)\beta_{2S}=\left(\frac{1}{s_{1}s_{2}}\right)\beta_{3S}$ (A-7)

As *β*_1S_ = *b*_1S_ *(*s*_1_/*s*_y_), *b*_2S_ = *b*_2S_ *(*s*_2_/*s*_y_) and *b*_3S_ = *b*_3S_ *(*s*_3_/*s*_y_), where *s_y_* is the standard deviation of *y*, the differential weighting is therefore

$\left(\frac{s_{1}}{s_{2}s_{3}}\right)b_{1S}+\left(\frac{s_{2}}{s_{1}s_{3}}\right)b_{2S}=\left(\frac{s_{3}}{s_{1}s_{2}}\right)b_{3S}$. (A-8)

Note that this constraint is also a general, irrespective of the number of PLS components extracted. For example, in Table 3, the one-component PLS regression coefficients for *Age*, *Cohort*, and *Period* on *SBP* in men are 0.083, -0.165 and -0.200. It can be verified that:

$\left(\frac{2.037}{6.882\times6.142}\right)0.083+\left(\frac{6.882}{2.037\times6.142}\right)\left(-0.165\right)\approx\left(\frac{6.142}{2.037\times6.882}\right)\times\left(-0.200\right)$.

The small inconsistency is due to rounding errors. Thus, PLS makes an implicit constraint to achieve a unique solution to the age-period-cohort analysis, and this knowledge is essential for the interpretation of the results from PLS.

In summary, the total effect, i.e. $\hat{\mathbf{y}}$ in equation (A-4), is unique, and PLS partitions the total effect of *Age*, *Period* and *Cohort* on *SBP* according to the relationships between *SBP* and the three covariates and the relationships amongst the perfectly collinear covariates by imposing an implicit constraint on the relationship amongst the regression coefficients.^42^ We call the constraint implicit or inherent because this constraint is not intentionally imposed by the PLS algorithms; instead, the constraint arises from the intrinsic mathematical relationship amongst the perfectly collinear covariates. We feel that this constraint in PLS analysis is both interpretable and justifiable. For instance, since *x*_1_ + *x*_2_ = *x*_3_, it seems quite reasonable to assume that the sum of the effects of *x*_1_ and *x*_2_ should be equal to the effect of *x*_3_. When the variances of the three variables are not equal and the variances do not reflect the relative importance of covariates, we give differential weighting to the constraint. This is why in this study, covariates are scaled in PLS regression.

For the nonlinear analyses in this study, where polynomial spline terms were used to model the nonlinear relationships, PLS regression coefficients for those polynomial terms were not affected by the collinearity amongst the linear functional terms. For example, when one of *Age*, *Period* and *Cohort* is removed from the model, this will not change the regression coefficients for the polynomial terms. This is because the identification problem is local to the linear terms, and this does not affect the estimation of the polynomial terms.^27-29^
